# Inflammatory or Reparative? Tuning Macrophage Polarization Using Anodized Anisotropic Nanoporous Titanium Implant Surfaces

**DOI:** 10.1002/smsc.202400211

**Published:** 2024-09-17

**Authors:** Ho‐Jin Moon, Karan Gulati, Tao Li, Corey Stephen Moran, Sašo Ivanovski

**Affiliations:** ^1^ Department of Dental Materials School of Dentistry Kyung Hee University Seoul 02447 Republic of Korea; ^2^ School of Dentistry and Oral Health Griffith University Gold Coast QLD 4222 Australia; ^3^ School of Dentistry The University of Queensland Herston QLD 4006 Australia; ^4^ Centre for Orofacial Regeneration, Reconstruction and Rehabilitation (COR3) School of Dentistry, The University of Queensland Herston QLD 4006 Australia; ^5^ Department of Prosthodontics School of Stomatology Capital Medical University Beijing 100050 P. R. China

**Keywords:** anodization, dental implants, macrophage polarization, macrophages, nanopores, titanium

## Abstract

Modulating macrophage phenotype based on implant surface characteristics, including topography and chemistry, has been employed to enhance osseointegration and long‐term functional outcomes for titanium (Ti)‐based implants. An excessive and/or prolonged M1 macrophage response can lead to damaging immune‐inflammatory reactions, negatively influencing the fate of the implant, and hence, modulating these responses via nanoscale implant surface modification is an emerging paradigm. Herein, an anodized titanium implant surface based on single‐step electrochemical anodization, with preserved underlying microfeatures and superimposed nanopores (50 and 70 nm), compared with irregular rough and microrough (machined‐like) surfaces, is investigated for its effect on the functions of primary macrophages in vitro. Significantly reduced macrophage proliferation and increased tissue‐reparative M2 phenotype polarization are confirmed for the nanopores, which are more pronounced for 70 nm diameter. Moreover, osteoclastogenesis is reduced while osteogenic differentiation of osteoblasts is enhanced for the nanopores (higher for 70 nm pores). Advanced nanoengineered Ti implants can enhance titanium implant tissue integration by modulating the inflammatory response at the implant–cell interface.

## Graphical abstract

This study presents a novel approach to enhancing titanium implant osseointegration by modulating macrophage phenotypes through nanoscale surface modification. It is demonstrated that anodized titanium implants with 70 nm nanopores, created via a single‐step electrochemical anodization, significantly reduce macrophage proliferation, promote a tissue‐reparative M2 phenotype, reduce osteoclastogenesis and enhance osteoblast differentiation, highlighting their potential to improve implant‐tissue integration.

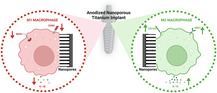

## 1 Introduction

Following the surgical insertion of biomedical devices, various cell types are involved in the healing cascade, leading to the integration of the implant, with macrophages playing a key role during the crucial early stages via the secretion of chemokines and cytokines.^[^ ^1^ ^]^ Macrophages are a highly heterogeneous cell population, spanning a spectrum of phenotypes between two broadly defined extremes of M1 (pro‐inflammatory) and M2 (pro‐reparative/regenerative) phenotypes.^[^ ^2^, ^3^ ^]^ The timely transition from an M1 (pro‐inflammatory) to an M2 (pro‐reparative) wound healing environment is essential for implant tissue integration. A persistent M1 response can lead to chronic inflammation and implant encapsulation with fibrotic tissue rather than functional integration.^[^ ^4^ ^]^ As a key initial wound healing event postimplantation, macrophage interaction with the implant surface depends on its physicochemical characteristics. Implant surface features, including size and topography, have been shown to influence immune‐inflammatory responses and macrophage functional polarization.^[^ ^5^ ^]^ Various topographical, chemical, and biological/therapeutic enhancements to conventional titanium (Ti) dental/orthopedic implants have been performed to achieve appropriate immunomodulation by facilitating the timely transition from M1 to M2 macrophage phenotype.^[^ ^6^, ^7^, ^8^ ^]^ Specifically, surface modifications of Ti (the most widely used implant material) facilitating nanoscale cell–matrix substratum interactions have been suggested to achieve desirable immunomodulatory functions.^[^ ^9^, ^10^, ^11^, ^12^, ^13^ ^]^

Among the various suggested nanoengineering strategies to modify Ti implant surfaces, electrochemical anodization (EA) has the advantages of scalability, cost‐effectiveness, and the ability to fabricate titanium oxide (TiO_2_) nanotubes/nanopores with great control over their characteristics.^[^ ^14^, ^15^, ^16^ ^]^ Nanotubes are like empty nanoscale test tubes (0.5–300 μm and 10–300 nm diameter) that self‐order on the surface of Ti upon anodization and have shown promising outcomes for enhancing bioactivity, osseointegration, and achieving controlled local release of therapeutics.^[^ ^17^ ^]^ Various studies report the influence of tube diameter, chemistry, and local release of anti‐inflammatory drugs from nanotube‐modified Ti implants toward achieving immunomodulatory functions.^[^ ^7^, ^11^, ^18^, ^19^, ^20^, ^21^, ^22^ ^]^ The effect of nanotube dimensions on macrophage function have also been investigated, with conflicting outcomes regarding the most optimum nanotube diameter.^[^ ^11^ ^]^ The varied observations may be attributed to: 1) various fabrication techniques/incorporation of electrolytes or improper cleaning of pores/tubes; 2) different internanotube distances or nanotube wall thicknesses; and 3) various sterilization procedures that alter surface chemistry.

In the domain of nanoengineered immunomodulatory implants based on anodized titanium with nanotubes, several research gaps remain unaddressed: 1) Most studies compare the immune functions of nanotubes with smooth/polished Ti and tissue culture plastic (TCP), both of which are irrelevant to the Ti orthopedic/dental implant market; 2) Typically, the microroughness of the Ti implant substrate is completely removed to enable easy fabrication of nanotubes. However, microroughness is currently regarded as the “gold standard” in achieving integration (especially in dentistry); 3) Inappropriate mechanical stability of the nanotubes can cause severe immunotoxicity; 4) Primary macrophage responses may differ from cell lines, which are routinely used in numerous studies; and 5) While local elution of nonsteroidal anti‐inflammatory drugs seem promising in vitro,^[^ ^23^, ^24^, ^25^ ^]^ the inability to gauge their effective concentration in vivo inside the bone microenvironment can raise concerns over high concentrations released locally from nanotubes.

An ideal immuno‐informed Ti implant surface would involve mechanically robust modification, preserving underlying microroughness, and modulating primary macrophage responses toward a timely transition to a regenerative macrophage (M2) phenotype. Interestingly, such anodized nanostructures have not been appropriately explored toward immunomodulation of Ti‐based bone/dental implants. More recently, our group has optimized EA for fabricating stable dual microrough and nanoporous surfaces on complex implant surfaces, including dental implant screws and abutments.^[^ ^26^ ^]^ Nanopores are nanotubes fused at the top (with underlying nanotubular structures) and are mechanically robust compared to well‐researched conventional nanotubes fabricated via anodization on Ti.^[^ ^27^, ^28^ ^]^ These structures also demonstrated “selective bioactivity” by reducing macrophage proliferation while maintaining the functions of fibroblasts and osteoblasts in vitro.^[^ ^29^ ^]^ Furthermore, these nanopores enabled fibroblast^[^ ^30^ ^]^ and osteoblast^[^ ^31^ ^]^ alignment, indicating strong mechanical stimulation, while macrophage morphology showed an inactive state. To summarize, nanopores offer the same surface chemistry and nanoscale topography as nanotubes, can be fabricated in one‐step EA with preserved underlying microroughness and retain drug loading/releasing functionality.

**Figure 1:**
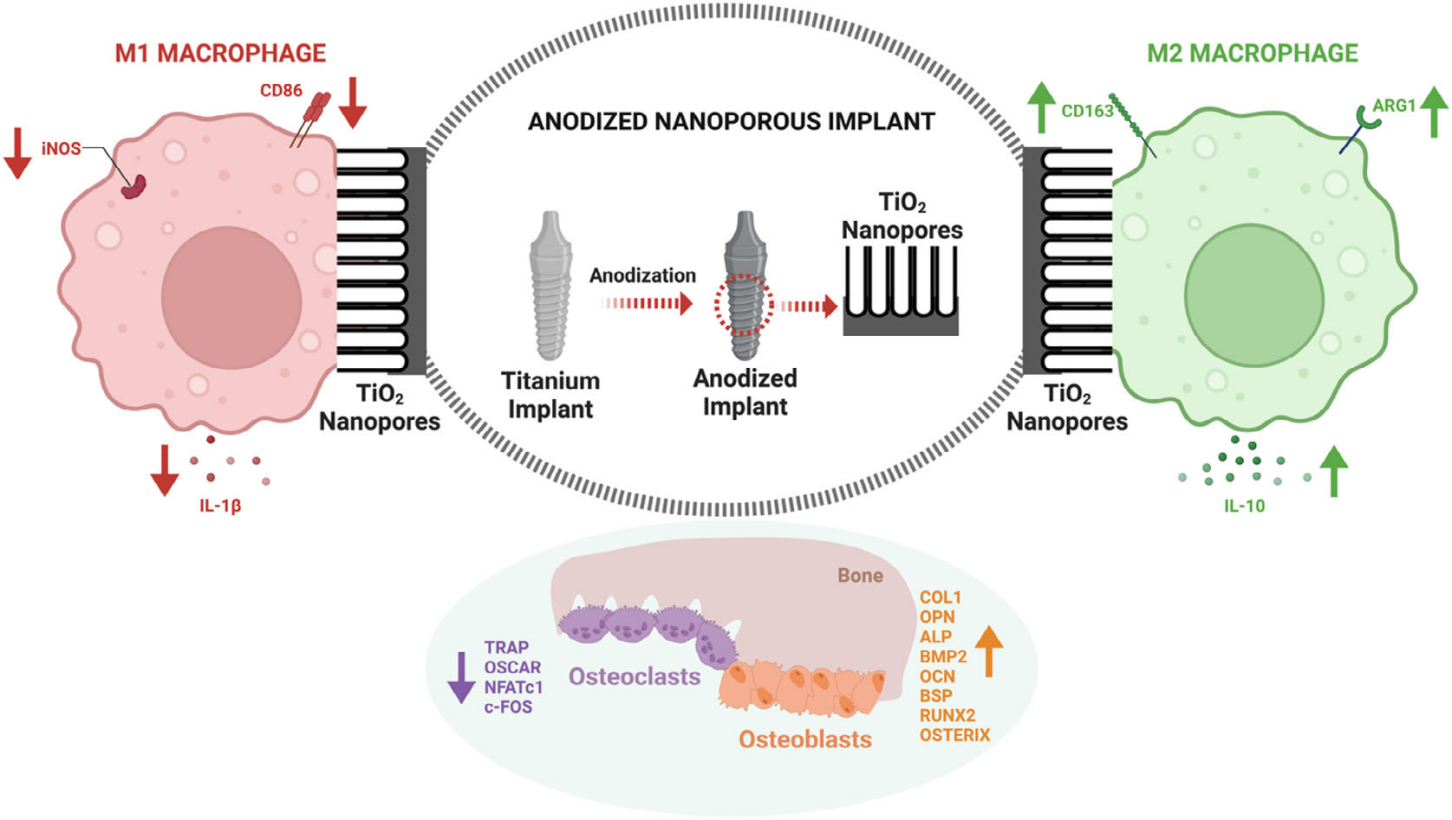
Immunomodulatory anodized nanoporous implants. Schematic representation of anodized nanoporous Ti implants and their influence on M1/M2 macrophages and osteoclast/osteoblast activity.

In this study, we fabricate nanopores of different dimensions on microrough Ti using a single‐step EA procedure to yield dual microrough and nanoporous substrates.^[^ ^14^ ^]^ The effect of these nanotopographies is evaluated on primary macrophage functions: proliferation, adhesion, and spreading; M1/M2 phenotype polarization; osteoclast and osteoblast differentiation, comparing them with clinically relevant implant surfaces as controls (**Figure** **1**). The findings from the study will contribute to the development of an optimized, readily translatable nanoengineered Ti implant surface toward immunomodulation and long‐term implant success in a bone/dental implant setting.

## 2 Results and Discussion

### 2.1 Anodized Titanium and Macrophage Adhesion

**Figure 2:**
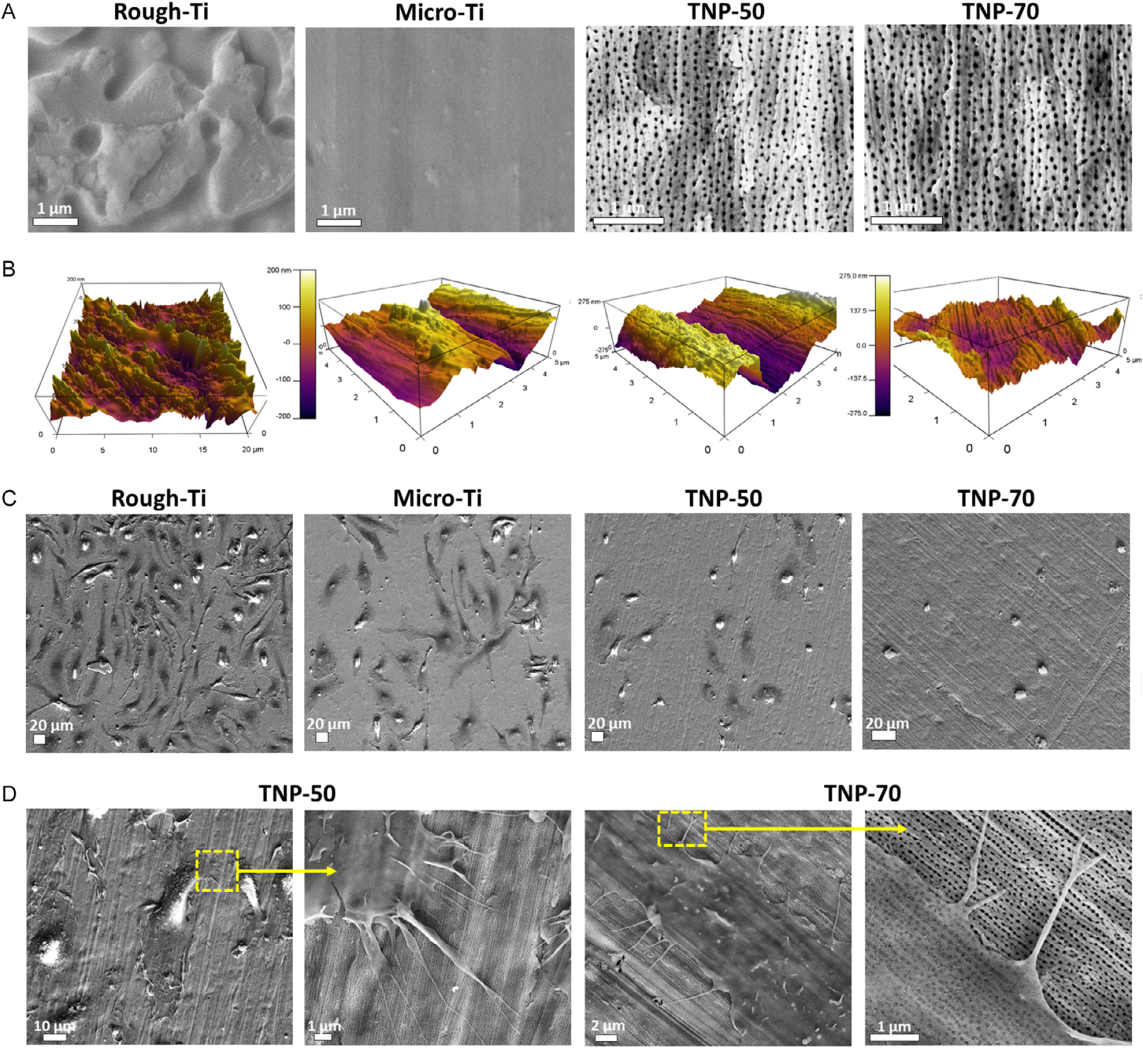
Surface topography and macrophage adhesion. A) Top‐view SEM images showing the surface topography of the implants. TNP: titania nanopores (50 and 70 nm diameter: TNP‐50 and TNP‐70). B) AFM images presenting the surface roughness. C) SEM images showing adhesion and spreading of macrophages on various implant surfaces (day 3) and D) high‐magnification of macrophage spreading morphology on nanopores (day 3).

Surface topography and roughness of the various substrates as imaged by SEM/AFM are presented in **Figure** **2**A,B. Rough‐Ti has very random roughness, while unidirectional mechanical preparation resulted in reduced roughness (resembling micromachining lines) in Micro‐Ti. Anodization was performed under controlled conditions with preconditioned (aged) electrolyte, which in a single‐step procedure enabled “conservation” of the underlying microroughness of Micro‐Ti and superimposed nanoporous structures (TNPs).^[^ ^26^ ^]^ Nanopores are like nanotubes fused at the top, with a nanotubular layer underneath. Our group has pioneered the optimized fabrication of such dual micro–nano structures on complex implant structures, including dental implant screws and abutments.^[^ ^26^, ^32^ ^]^ Microroughness is regarded as the “gold standard” for ensuring initial implant integration in clinical dentistry. Here, aligned with the underlying microscale lining (crest‐and‐trough‐like microarchitecture), nanopores self‐ordered in an anisotropic fashion. Our previous studies thoroughly evaluated TNPs’ characteristics, including surface topography, roughness, hydrophilicity, chemistry, crystallinity, and mechanical stability.^[^ ^14^, ^26^, ^27^, ^31^, ^33^ ^]^ We have recently shown that primary human gingival fibroblasts and osteoblasts align parallel to these microscale nanopores, corresponding to a strong mechanotransduction effect.^[^ ^31^, ^34^ ^]^

Interestingly, most EA attempts at modifying the implant's surface involve smooth surfaces or multistep anodization. Ensuring easy translation, our optimized procedure is single step and cost‐effective and can easily be extended to the broader titanium‐based implant market, as well as zirconium and other metallic implants.^[^ ^35^, ^36^ ^]^ Furthermore, these nanopores outperform conventional titania nanotubes (TNTs) concerning mechanical stability, mainly attributed to no gap between individual nanotubes and fabrication under controlled conditions with aged electrolytes.^[^ ^27^, ^28^ ^]^

Various studies have suggested that surface topography overrides chemistry in initial cellular contact (6–48 h).^[^ ^37^ ^]^ Interestingly, most studies focusing on advancing bioactivity of dental implants center on soft‐ and hard‐tissue integration; however, the first cells to race at the implantation site are immune cells. Indeed, it is well established that the adhesion and proliferation of macrophages regulate the inflammatory response.^[^ ^38^ ^]^ Consistent with our previous reports, current macrophage proliferation data (Figure S1, Supporting Information) confirm significantly reduced proliferation rates for BMM on the TNP‐70 surface.^[^ ^29^ ^]^ This finding also correlated with other reports with nanotubes, whereby 60–70 nm range diameters showed the most immunomodulatory effects.^[^ ^11^, ^39^ ^]^ Interestingly, more mechanically robust nanopores with preserved underlying microroughness have not been explored appropriately toward immunomodulation. This study aimed to address this challenge and showcase data comparing nanotopography and microtopography (with similar microscale features).

Results of the BMM live/dead assay (Figure S2, Supporting Information) indicated a reduced number of cells attached to TNP‐70 in the absence of cell death. As such, there is no toxic effect of TNP surfaces upon the cultured macrophages; rather they are an impediment to macrophage adhesion and proliferation. Sharing the same chemistry as nanotubes, nanopores have maintained the activity of fibroblasts and osteoblasts.^[^ ^29^ ^]^ The proliferation and live/dead observations can be attributed to various nanoporous characteristics, including roughness, chemistry, and mechanical stimulation.^[^ ^40^, ^41^, ^42^, ^43^ ^]^ Additionally, a 20 nm pore size difference between TNP‐50 and TNP‐70 and related changes in other parameters, including roughness may account for the significant reduction in proliferation for TNP‐70.^[^ ^29^, ^43^ ^]^

The morphology of macrophage spreading (day 3 of the culture), presented as top‐view SEM, is shown in Figure 2C. A maximum number of cells spreading well onto the surface can be seen for Rough‐Ti, in contrast to Micro‐Ti, where there are comparatively fewer spreading cells. However, for nanopores, BMM numbers are fewer still and show drastically reduced cellular adhesions and spreading. Indeed, for TNP‐70, BMM numbers are very sparse, indicating that the surface features were able to reduce macrophage adhesion significantly. Anodized titanium surfaces demonstrated significantly reduced macrophage adhesion compared to nonanodized titanium. This reduction may be attributed to the surface property alterations induced by the anodization process. The trend of decreased macrophage adhesion on nanotopographies aligns with previous research, which reported reduced macrophage adhesion on 40–70 nm diameter of TNTs compared to conventional surfaces.^[^ ^42^, ^44^, ^45^, ^46^ ^]^ Further, studies have indicated that surfaces with microrough features tend to improve the adhesion of macrophages and increase their secretion of inflammatory cytokines, such as IL‐1β, IL‐6, and nitric oxide, compared to smooth surfaces.^[^ ^47^ ^]^ Notably, macrophages are myeloid cells, do not interact with the ECM via stress fiber formation, and rely on short‐lived focal complexes and podosomes to achieve phagocytosis and migration.^[^ ^48^ ^]^

The more “oval” spreading (and lack of significant cell stretching) morphology of BMM on nanopores represents a more inactivated state, as observed for nanostructured Ti implants.^[^ ^49^, ^50^ ^]^ For activated macrophages, an extensive spreading morphology with increased surface area and prominent protrusions (filopodia) are expected, which are not present for the TNPs. Based on their morphology, migration modes of macrophages can also be defined, which correspond to the physical and chemical cues of the environment. Based on previous reports, a mesenchymal mode (elongation with many long protrusions) for BMM on Micro‐ and Rough‐Ti and an amoeboid‐migration mode (spherical cells and reduced short protrusions) on TNPs are seen.^[^ ^51^, ^52^ ^]^ In the current study, high‐magnification SEM revealed that BMM on TNPs did not correspond to underlying nanopore alignment and are randomly organized (Figure 2D), in contrast to the previously reported directional alignment of gingival fibroblasts and osteoblasts with the underlying nanopores indicating strong mechanical influence.^[^ ^31^, ^53^ ^]^

Many investigations have suggested that pore size is a crucial determinant of biomaterials’ success via its influence on cell adhesion, nutrient exchange and, ultimately, cell functioning.^[^ ^38^, ^54^, ^55^, ^56^ ^]^ Garg et al. pointed out that a natural and spreading macrophage morphology, especially in a 3D microenvironment, may direct the macrophages toward homeostatic reparative roles (as performed by M2 in native tissue).^[^ ^57^ ^]^ For biomaterials with a smaller pore architecture, whereby the macrophages may be primarily concentrated onto the surface alone (with no infiltration like porous counterparts), their attempt to invade results in a “frustrated phagocytosis” and M1 tissue destructive roles.^[^ ^57^ ^]^ Besides surface topography, chemical moieties on the surface of nanopores, including TiF6 complexes (incorporated from anodization electrolyte), can also cause an immunomodulatory effect. However, whether such minute concentrations influence macrophage activity significantly is still unexplored. Our findings align with reports for anodized Ti with TiO_2_ nanotubes and the diameter range that successfully reduced macrophage activity.^[^ ^39^, ^58^ ^]^ In addition, it is noteworthy to mention that nanopores retain the ability to load/release therapeutics and hence can be used toward local elution of nonsteroidal anti‐inflammatory drugs, as previously demonstrated for nanotubes.^[^ ^53^, ^59^, ^60^, ^61^, ^62^ ^]^

### 2.2 Nanotopography Induced M1/M2 Polarization of M0 Macrophages

**Figure 3:**
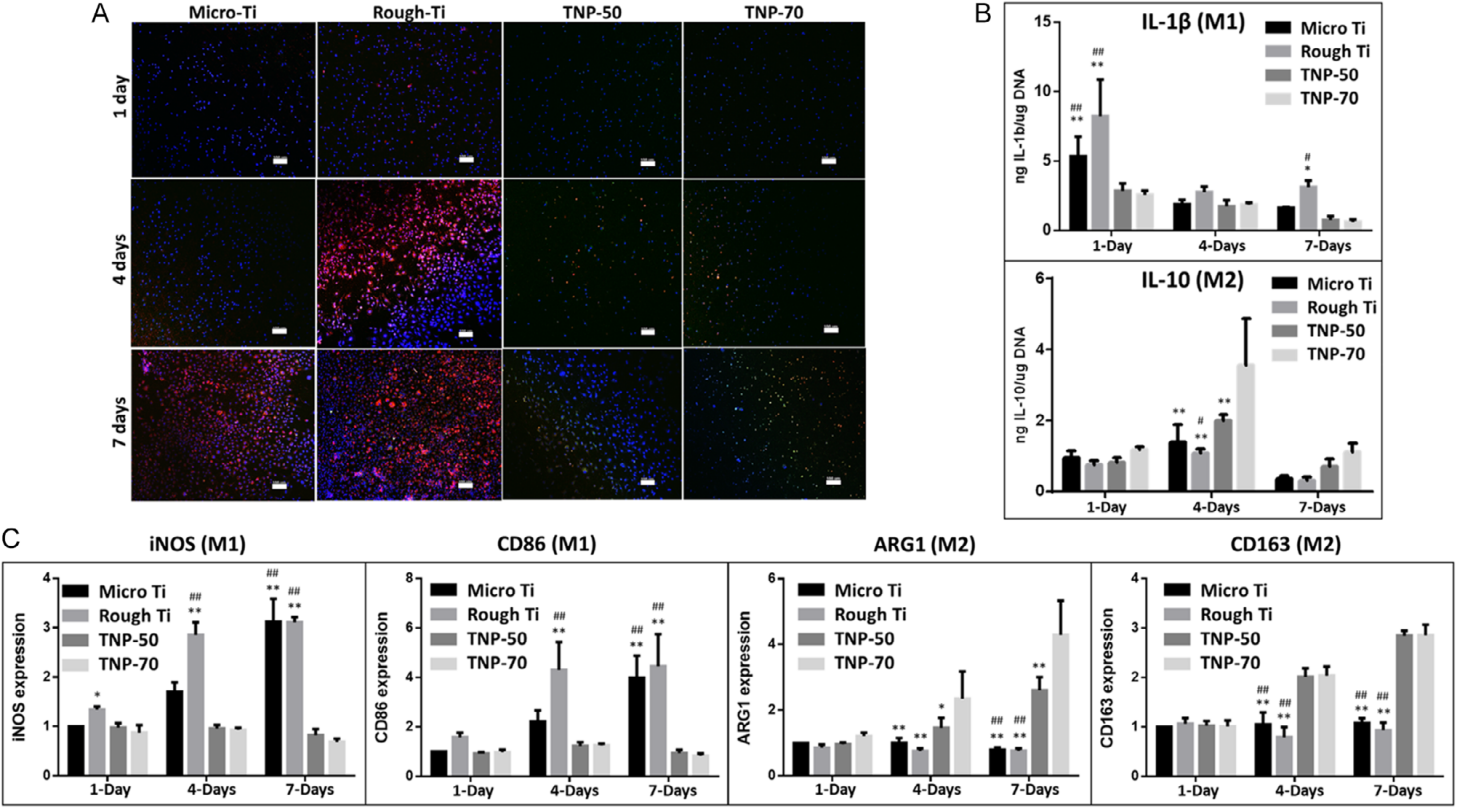
M1/M2 macrophage phenotypes with M0 macrophages. A) Confocal imaging of immunofluorescence staining for macrophages (M0) showing expression of M1 (CD11c: red) and M2 (CD206: green) markers at 1, 4, and 7 days on various implants [DAPI: blue, scale bar = 100 μm]. B) Concentration of anti‐inflammatory cytokines (determined by ELISA) released after 1, 4, and 7 days of culture on various Ti substrates: IL‐1β (M1) and IL‐10 (M2). C) Real‐time PCR expression of M1 [iNOS and CD86] and M2 [Arg1 and CD163] markers. **p < 0.01; *p < 0.05 significantly different from TNP‐70. ^##^ *p* < 0.01, ^#^ *p* < 0.05 significantly different from TNP‐50. TNP: titania nanopores (50 and 70 nm diameter: TNP‐50 and TNP‐70).

Immunofluorescence imaging was used to assess macrophage polarization on days 1, 4, and 7 of culture, using staining for CD11c (Red, M1) and CD206 (Green, M2) as presented in **Figure** **3**A. Limited polarization was observed on day 1; however, a difference between the various Ti substrates can be seen from day 4 onward. On day 4, more significant M1 staining was seen for Rough‐Ti than other substrates. M1 staining at day 7 is visible for Micro‐ and Rough‐Ti, whereas the macrophages on TNPs are predominantly M2‐stained. Furthermore, a prominent M2 staining between the nanopores is seen for the 70 nm nanopores (TNP‐70) compared to 50 nm.

In line with the T‐helper cell nomenclature, macrophages are classified into two phenotypes: M1 (pro‐inflammation) and M2 (pro‐healing).^[^ ^63^, ^64^ ^]^ Modulating macrophage proliferation toward attenuated foreign body response (FBR) can be achieved by nanostructuring of the implant surface.^[^ ^38^, ^65^, ^66^ ^]^ Enzyme‐linked immunosorbent assay (ELISA) was used to estimate protein levels of the proinflammatory (M1) and anti‐inflammatory (M2) cytokines IL‐1β and IL‐10, respectively (Figure 3B). It is established that IL‐1β expresses a biphasic response (high levels postinjury becoming undetectable within 3 days^[^ ^67^ ^]^) and behaves as a “double‐edged sword” by increasing bone formation or resorption depending on its concentration.^[^ ^68^, ^69^ ^]^ Moreover, IL‐1β binds to IL‐1 R/Toll‐like receptors and leads to nuclear factor kappa B activation, essential for osteogenesis and osteoclastogenesis.^[^ ^70^ ^]^ Additionally, IL‐1β inhibits collagen synthesis and BMP‐2 activity in a dose‐dependent manner.^[^ ^71^, ^72^ ^]^ We saw that for both the nanoporous substrates (compared to Micro‐ and Rough‐Ti), there was a significant reduction in IL‐1β secretion on day 1. In contrast, after 4 days, the reduction was again seen for nanopores (compared with Rough‐Ti). Following previous studies, this prominent IL‐1β decline for the TNPs translates to improved bone healing.^[^ ^49^ ^]^ Upregulation of the M2‐marker IL‐10 was seen on day 4 for TNP‐70, while no significant differences between substrates were observed by day 7.

M1 macrophages produce IL‐1β, IL‐6, inducible nitric oxide synthase (iNOS), and TNF‐α with surface markers CD86 and CCR7, while M2 macrophages produce arginine 1 (Arg1) and IL‐10 with surface markers CD163 and CD206.^[^ ^73^ ^]^ In the current study, we looked at the expression of M1 markers iNOS and CD86 (iNOS) and M2 markers Arg1 and CD163 (Arg1), as presented in Figure 3C. For the nanoporous substrates, reduced expression of iNOS and CD86 in M1 cells is seen on both day 4 and day 7. It is known that macrophages and neutrophils release enzymes (including nitric oxide synthase: iNOS) to degrade/phagocytose the implanted biomaterial.^[^ ^74^ ^]^ It has also been reported that IL‐10 (and TGF‐β, IL‐4) provides inhibitory signals to iNOS, which correlates with the upregulation of IL‐10 and downregulation of iNOS seen in the present study for TNPs.^[^ ^75^ ^]^ In contrast, a significant upregulation in the expression of the M2 markers Arg1 and CD163 was seen for the TNPs.

Our findings indicate that nanopores promote a shift toward an M2 (pro‐healing) phenotype in BMM in vitro. Surface characteristics, including wettability, charge, and topography, greatly influence the behaviors of immune cells.^[^ ^76^ ^]^ Published reports suggest that smoother Ti surfaces induce M1 activation, while hydrophilic rough Ti surfaces induce M2 activation.^[^ ^40^, ^77^, ^78^, ^79^ ^]^ In this context, the current study's nanopores are more hydrophilic than other implant substrates, and as they preserve the underlying microroughness, they are rougher compared to conventional nanotubes.^[^ ^31^, ^43^ ^]^

### 2.3 Nanotopography‐Induced Polarization of M1 and M2 Macrophages

**Figure 4:**
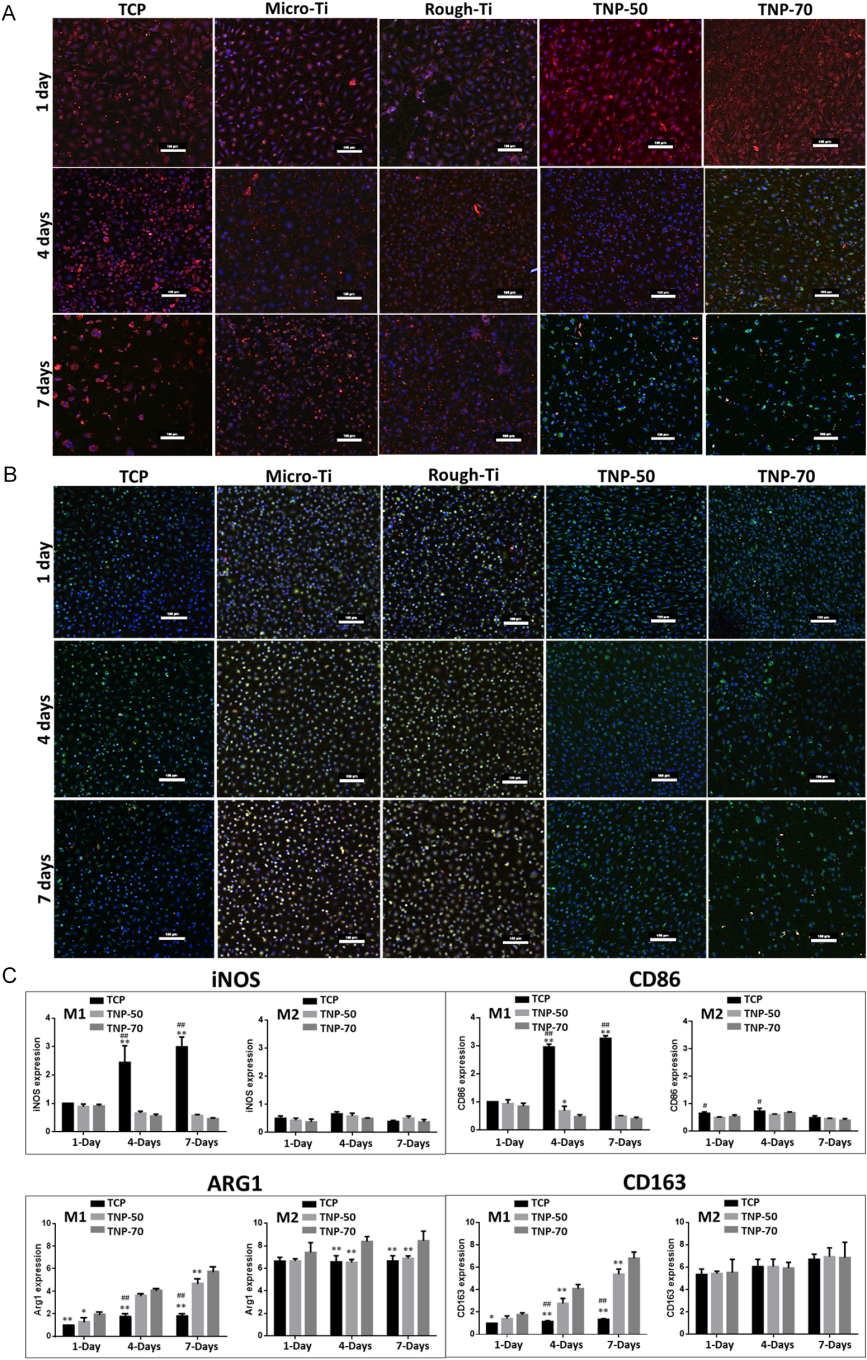
M1/M2 macrophage phenotypes with M1 and M2 macrophages. A) Confocal imaging of immunofluorescence staining of M1 macrophages showing expression of M1 (CD11c: red) and M2 (CD206: green) markers at 1, 4, and 7 days on various implants [DAPI: blue, scale bar = 100 μm]. B) Confocal imaging of immunofluorescence staining of M2 macrophages showing expression of M1 (CD11c: red) and M2 (CD206: green) markers at 1, 4, and 7 days on various implants. Scale bar = 100 μm. C) M1 and M2 macrophage gene expression of CD86, iNOS, CD163, and Arg1 with M1/M2 macrophages on aligned TiO_2_ nanopores. **p < 0.01, *p < 0.05 significantly different from TNP‐70. ^##^ *p* < 0.01, ^#^ *p* < 0.05 significantly different from TNP‐50. TNP: titania nanopores (50 and 70 nm diameter: TNP‐50 and TNP‐70).

We determined the effect of our various Ti substrates on the phenotype of M1 (LPS‐stimulated M0) and M2 (IL‐4‐ and IL‐13‐stimulated M0) macrophages, assessed by confocal immunofluorescence staining (**Figure** **4**A,B). Our data show that TNPs promoted a shift toward an M2 phenotype (green CD206 marker) in M1 BMM from day 4, with the effect most significant by day 7. The M2 phenotype in IL‐4/IL‐13‐stimulated BMM was preserved (green CD206 marker) for all substrates at various time points, indicating surfaces conducive toward M2 polarization. We further investigated gene expression of CD86, iNOS, CD163, and Arg1 with M1/M2 macrophages on nanopores, compared with TCP (Figure 4C). For M1, downregulation of iNOS and CD86 at days 4 and 7 was seen for the TNPs.

Moreover, for M1, TNP‐70 showed increased expression of Arg1 for all time points compared with both TNP‐50 and TCP. Similarly, for M1, CD163 expression was increased significantly for TNP‐70 from day 4. Conversely, for M2, while iNOS and CD163 expression did not change significantly, CD86 was slightly downregulated for TNPs. Following the trend of M1, Arg1 expression for M2 was enhanced considerably for TNP‐70 from day 4, compared to TNP‐50 and TCP substrates. Overall, the findings show that TNPs promote a more pro‐healing phenotype (M2) even for M1/M2 induced macrophages, with the effect prominent for TNP‐70 nanopores. This also confirms that a 20 nm difference between nanopore diameters can result in differences between rates of macrophage polarization. It is established that prolonged M1 activation can cause tissue injury, and hence, facilitating the transition from M1 into M2 can favor tissue healing and remodeling.^[^ ^57^ ^]^

Modulating cell functions based on varying biomaterial pore size is a crucial determinant widely explored for various biomaterial types and applications.^[^ ^80^ ^]^ In our study, TNPs produced promising evidence for enabling the transition of proinflammatory M1 macrophages to the pro‐healing M2 phenotype (statistically higher Arg1 expression), with the effect more prominent for the TNP‐70. It has been reported that microscale cues may direct macrophages toward a 3D orientation, acquiring a natural morphology, and hence, M2‐like reparative roles.^[^ ^57^, ^81^ ^]^ Applicable to both a dental and orthopedic implant setting, our dual microrough and nanoporous TNP‐70 modification can promote the M2 phenotype toward implant healing and integration. This effect may be crucial in patients with ongoing conditions such as poor bone quality/quantity or bacterial infection.^[^ ^82^ ^]^ It is worth noting that the exact mechanisms contributing to mediating immune responses via nanotopography are interdependent on multiple factors.^[^ ^83^ ^]^

### 2.4 Osteoclast Differentiation of Macrophages on Nanopores

**Figure 5:**
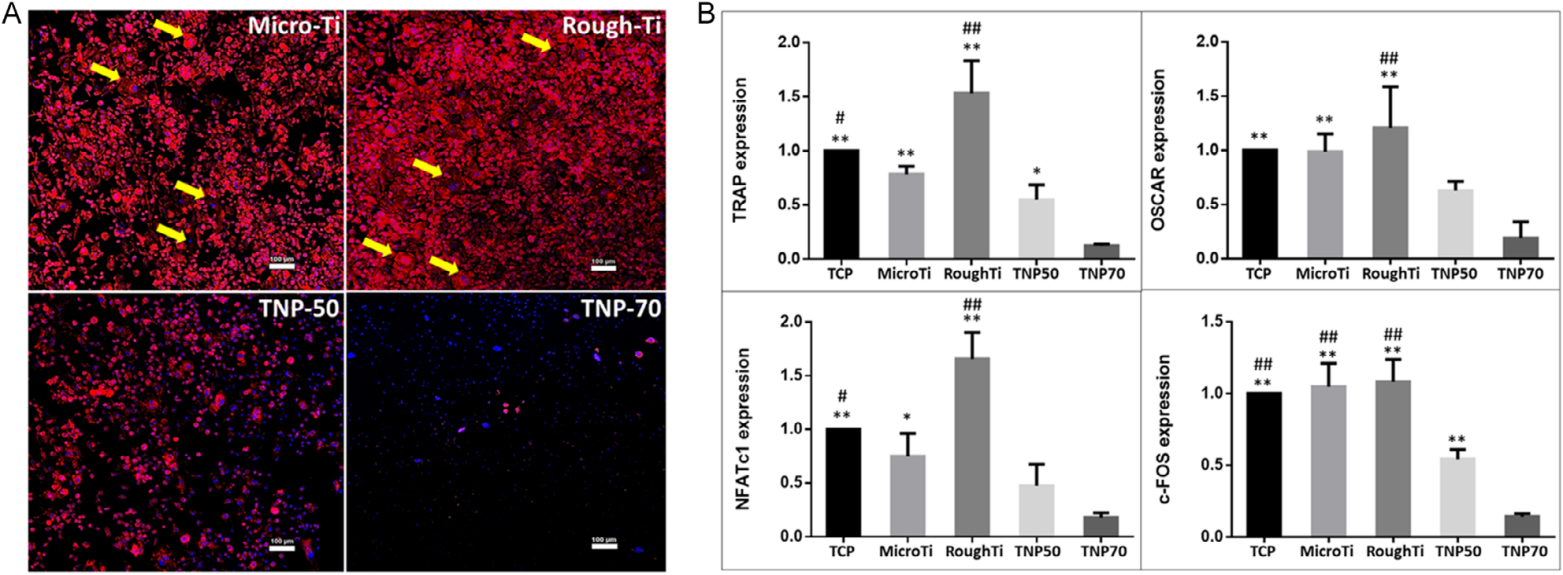
Osteoclast differentiation of macrophages (induced with RANKL + M‐CSF) on TNPs. A) F‐action ring staining (yellow arrows show differentiated osteoclasts, scale bar = 100 μm). B) Gene expression of NFATc1, c‐FOS, TRAP, and OSCAR for osteoclast differentiation. **p < 0.01, *p < 0.05 significantly different from TNP‐70. ^##^ *p* < 0.01, ^#^ *p* < 0.05 significantly different from TNP‐50. TNP: titania nanopores (50 and 70 nm diameter: TNP‐50 and TNP‐70).

Macrophages influence osteoclastogenesis and can differentiate into osteoclasts, inducing or inhibiting bone formation.^[^ ^84^, ^85^ ^]^ Macrophages are osteoclast precursors, and differentiation can be induced by RANKL (nuclear factor kappa‐B ligand) and M‐CSF.^[^ ^86^, ^87^ ^]^ Similarly, macrophages in the current study were differentiated into osteoclasts using RANKL and M‐CSF. As seen from the osteoclast staining data (**Figure** **5**A), almost negligible differentiated osteoclasts (multinuclear cells) can be seen on the nanopores. However, larger osteoclasts (pronounced actin ring staining) are visible on both Micro‐ and Rough‐Ti.

Gene expression of TRAP, OSCAR, NFATc1, and c‐FOS was assessed to determine the osteoclastic activity of macrophage cultures on our various Ti surfaces (Figure 5B). Our finding of significant osteoclast inhibition (overall suppression of osteoclastogenic gene expression) mediated by nanoporous surfaces compared to controls (most prominent for TNP‐70) supports the potential for nanoporous implants (TNP‐70) to inhibit osteoclast formation. The macrophage‐induced osteogenic or osteoclastic effects are believed to be attributed to the macrophage switch pattern instead of the specific phenotype.^[^ ^84^ ^]^

### 2.5 M0 Macrophage‐Induced Osteoblast Differentiation

**Figure 6:**
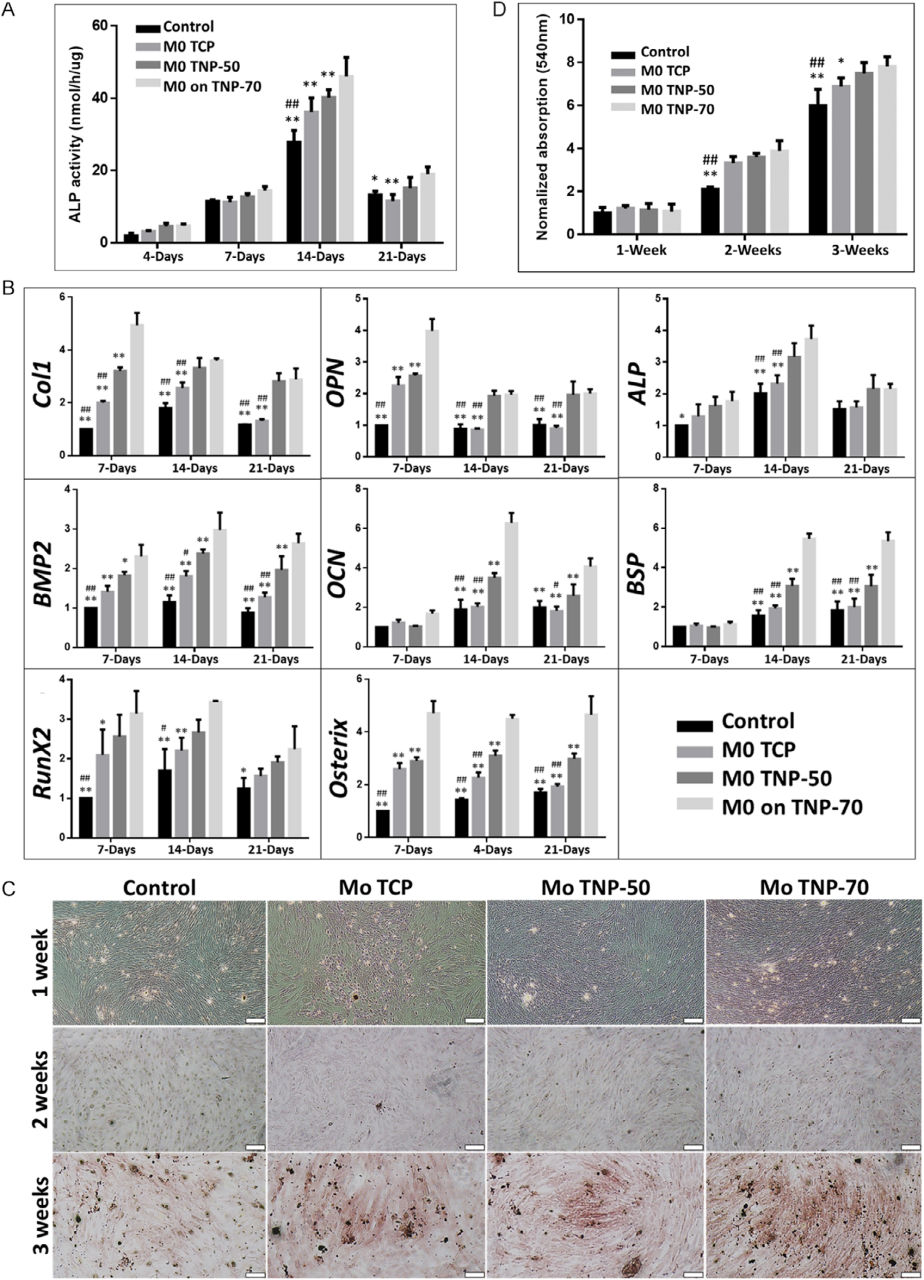
Osteogenic performance of MC3T3 cells cultured with M0 macrophage conditioned medium on TNP. A) ALP activity. B) RT‐PCR gene expression. C,D) Alizarin red staining. Control: only osteogenic media (without conditioned media) for co‐culture. Scale bar = 200 μm. **p < 0.01, *p < 0.05 significantly different from Mo TNP‐70. ^##^ *p* < 0.01, ^#^ *p* < 0.05 significantly different from Mo TNP‐50. TNP: titania nanopores (50 and 70 nm diameter: TNP‐50 and TNP‐70).

**Figure** **6**A shows the ALP activity for the various substrates in M0‐conditioned media. ALP activity is crucial for bone mineralization and represents a useful biochemical marker for bone formation. From 2 weeks, significantly enhanced ALP activity is seen for M0‐TNP‐70 compared to all other surfaces. Similarly, M0‐TNP‐50 showed increased activity compared to the control (osteogenic media without conditioned media) at 2 weeks. In vivo investigations have established that reduced macrophage number or complete exhaustion corresponds to impaired or delayed bone healing, suggesting the significance of macrophage functions in osteogenesis.^[^ ^85^, ^88^, ^89^ ^]^ To gauge the osteo‐immunomodulatory capacity of nanoporous implants, we investigated the expression of osteogenic factors after culturing MC3T3 cells with an M0 macrophage‐conditioned medium (Figure 6B). The findings indicated that for the nanoporous substrates, a significant elevation in expression of Col1, OPN, BMP‐2, RunX2, and osterix was observed for all time points (1, 2, and 3 weeks), with TNP‐70 showing the more favorable osteogenic response.

Additionally, similar enhancements were more significant for OCN and BSP from 2 weeks onward. ALP expression was significantly increased at the 2 week time‐point for nanoporous Ti. We further investigated the osteogenic performance of MC3T3 cells cultured on nanopores in an M0 macrophage‐conditioned medium, with alizarin red staining revealing more mineralization nodule formation by MC3T3 cells on the nanoporous surfaces (Figure 6C,D). In line with the previous parameters examined, more nodule formation was evident on TNP‐70. This, together with our previous data demonstrating that M0 macrophages most effectively switched to M2 on TNP‐70 and the efficacy of cytokines released by M0 macrophages on TNP‐70 for osteogenesis, strongly supports favorable effects of nanoporous architecture on osteogenesis, and in particular the effectiveness and suitability of the TNP‐70 surface for eliciting an immunomodulatory response to induce osteoblast differentiation.

### 2.6 Macrophage Polarization and Osteoblast Differentiation

Implant nanotopography directly influences the orchestration of osseointegration, and considering the role of macrophages in bone regeneration/remodeling, the modulation of macrophages to achieve osteogenesis is attracting attention.^[^ ^90^ ^]^ It is established that M2 macrophages can recruit osteoblasts via the secretion of cytokines (IL‐10, BMP‐2, and TGF‐β) to augment osteogenesis and achieve new bone formation around implants.^[^ ^91^, ^92^ ^]^

**Figure 7:**
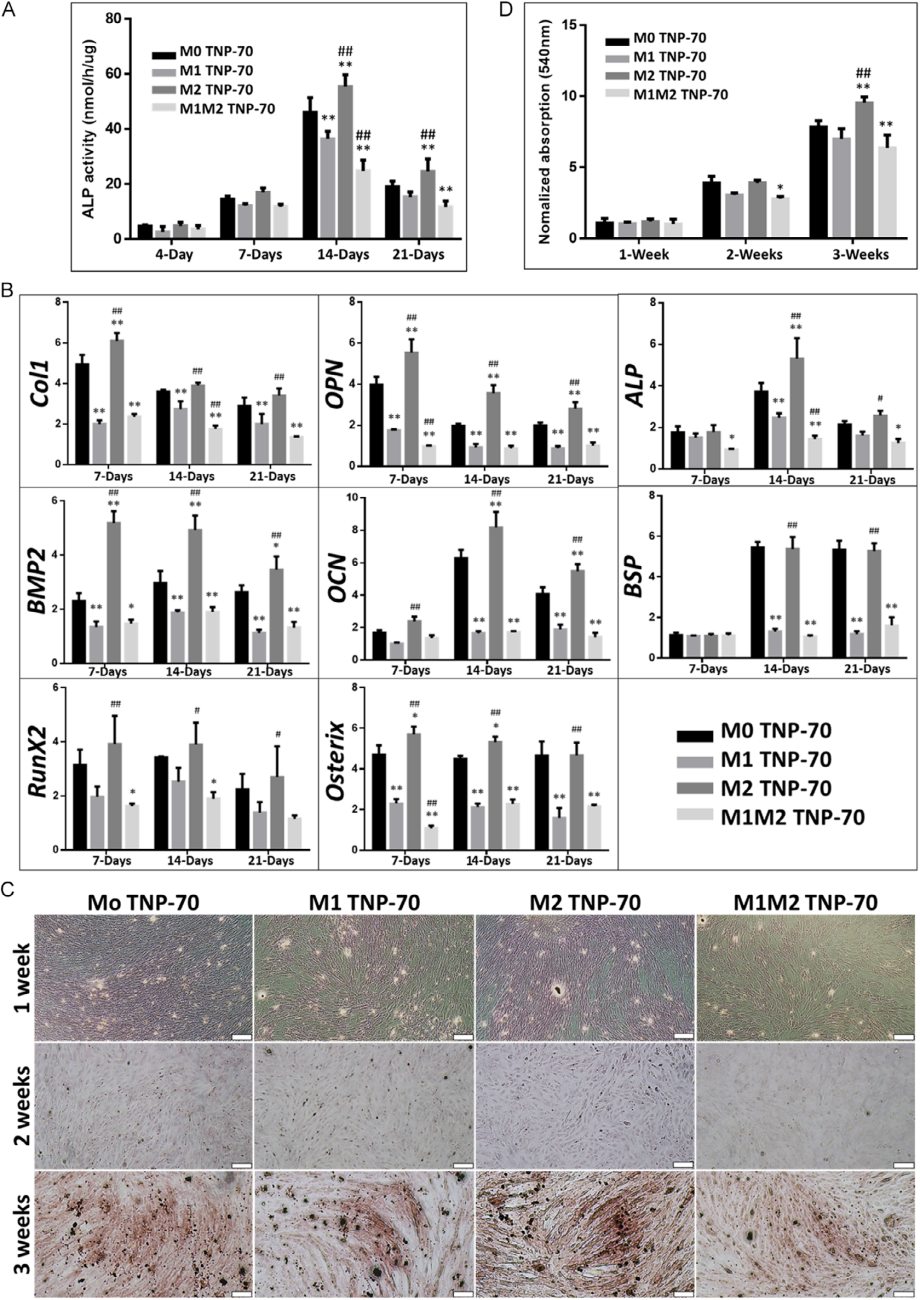
Osteogenic differentiation of MC3T3 cells cultured with various macrophage phenotype (M0, M1, M2, M1 induced‐M2 macrophage) conditioned medium on TNPs. A) ALP activity. B) RT‐PCR gene expression. Alizarin red staining of MC3T3 cells: C) optical images and D) quantitative analysis. Scale bar = 200 μm. **p < 0.01, *p < 0.05 significantly different from Mo TNP‐70. ^##^ *p* < 0.01, ^#^ *p* < 0.05 significantly different from M1 TNP‐70. TNP: titania nanopores (50 and 70 nm diameter: TNP‐50 and TNP‐70).

The results from previous sections confirmed that TNP‐70 could modulate M0 macrophages. We investigated the osteogenic performance of M1 and M2 macrophages on TNP‐70. M0, M1, and M2 macrophages were seeded on the TNP‐70 surface, and the influence of factors released from the cells on bone differentiation was examined. M0 macrophages were first induced to M1 and then M2 macrophages (M1M2 group). To gauge the influence of Ti nanotopography on osteogenic differentiation, we analyzed the ALP activity of MC3T3 cells (**Figure** **7**A). Upon supplementation of macrophage cytokines, the results show that M2 TNP‐70 exhibited the highest ALP activity at 14 and 21 days of culture. It is noteworthy that ALP is an early marker for osteogenic differentiation;^[^ ^28^, ^63^ ^]^ the results confirm that M2 macrophage cytokines enhanced media on TNP‐70 induces osteogenic differentiation of MC3T3 cells.

Expression of osteogenic‐related genes, COL 1, OPN, ALP, BMP‐2, OCN, BSP, RUNX2, and osterix was performed and the results are presented in Figure 7B. It revealed that across all time points studied, M2 TNP‐70 outperformed others in exhibiting significantly augmented expression for COL 1, OPN, BMP‐2, OCN, RUNX2, and osterix. For ALP and BSP, M2 TNP‐70 showed significantly enhanced expression at 14 and 21 days. Further, the optical images for alizarin red stained MC3T3 cells (Figure 7C) and quantitative analysis of alizarin red staining (Figure 7D) cultured on varied substrates with macrophage cytokines provide evidence that M2 TNP‐70 exhibited the highest extracellular matrix mineralization at 3 weeks.

Taken together, as compared to other groups, M2 macrophages on TNP‐70 exhibited an upregulated bone differentiation effect. Furthermore, bone differentiation was prominent in the group treated with M0 macrophages, compared with M1 on TNP‐70. Further, the M1M2 group had the least (or comparable to the M1 group) influence on bone differentiation. The data show that induction into M2 macrophages from M0, M1 was more effective in M0 macrophages. It is worth noting that M1M2 macrophages showed a lower osteogenic performance due to a significantly more significant effect of M1.

In summary, the current study revealed that a 20 nm pore difference in TiO_2_ nanoporous modification of Ti implants could enable immunomodulation toward a more reparative (M2 polarization of macrophages) function suited toward osseointegration. Specifically, TNP‐70 promoted upregulation of M2 polarization, reduced osteoclast, and enhanced osteoblast differentiation and mineralization. M2 macrophages create a more conducive healing environment compared to M1 macrophages by facilitating the transition from an inflammatory to a reparative environment. They secrete anti‐inflammatory cytokines and growth factors, enhancing angiogenesis and extracellular matrix formation, crucial for implant vascularization and integration with bone tissue.^[^ ^93^ ^]^ Early polarization of macrophages toward an M2 phenotype on anodized titanium surfaces can enhance bone healing and the development of a mature implant–bone interface, which is more resilient against microbial challenges during the crucial early postimplantation healing.^[^ ^94^, ^95^, ^96^ ^]^ Despite this, to ensure clinical translation of this nanoengineered Ti surface in dental/orthopedic implants, further investigations, especially in‐depth in vivo implantation aimed at evaluating optimized immunomodulatory titanium implants, are needed.

## 3 Conclusions

Appropriate immunomodulation from the surface of nanoengineered implants can reduce inflammation and FBR and initiate osteogenesis, translating into accelerated integration and enhanced long‐term survival. We investigated the performance of primary macrophages on anodized titanium with anisotropic TNPs, aiming to design the next generation of immuno‐responsive and osteogenic orthopedic and dental implants. The 70 nm diameter nanopores (TNP‐70) had a prominent positive influence on M2 polarization, which could mediate tissue repair via the release of pro‐healing cytokines. Further, TNP‐70 significantly downregulated osteoclast activity and differentiation, translating into a more favourable bone remodeling environment. TNP‐70 (with M2‐induced culture media) also exhibited significantly augmented bone differentiation properties. Tuning the crosstalk between macrophages, osteoclasts, and osteoblasts, this study showed that TNP‐70 is a promising surface modification strategy achieved via single‐step anodization of micromachined Ti implants that can orchestrate osteo‐immunomodulation. Further developments in this domain are needed via long‐term in vivo implantation models to confirm the clinical translation potential of these immunomodulatory nanoengineered implants.

## 4 Experimental Section

4.1

4.1.1

##### Surface Modification of Titanium

Ti flat foil (99.5% purity) with a thickness of 0.20 mm was obtained from Nilaco, Japan. All chemicals were obtained from Sigma–Aldrich, Australia and were used as received. The as‐received Ti foil (Rough‐Ti) was mechanically prepared (Micro‐Ti), followed by sonication in acetone and ethanol, as described previously.^[^ ^14^, ^33^ ^]^ Before further treatment, the foil was cut in 10 mm^2^ squares and sonicated in ethanol. Electrochemical anodization was performed on Micro‐Ti using the recently optimized protocol in a custom‐designed electrochemical cell, as described elsewhere.^[^ ^27^ ^]^ Briefly, anodization was performed inside appropriately aged ethylene glycol electrolyte (with 1% v/v deionized water and 0.3% w/v ammonium fluoride), using mechanically prepared target Ti foil (enclosed in an in‐house electrode) as anode and cleaned Ti foil as cathode, connected to the DC power supply (Keysight, Australia).^[^ ^26^ ^]^ Single‐step anodization was performed at 25 °C under constant magnetic stirring, using various voltages and times. 60 V 10 min yielded nanopores with ≈50 nm diameter (TNP‐50) and 80 V 15 min yielded nanopores with ≈70 nm diameter (TNP‐70). Postanodization, all substrates were cleaned in deionized water and air‐dried. Before further investigations, all substrates were sterilized using UV irradiation for 1 h on either side.^[^ ^97^ ^]^

##### Surface Characterization of Titanium Topography

The prepared substrates’ surface morphology was characterized using a field emission scanning electron microscope (Zeiss Sigma FESEM). Briefly, the substrates were mounted on an SEM holder with double‐sided conductive tape and coated with a 5 nm thick layer of platinum. Images with a range of scan sizes at normal incidence and at a 30° angle were acquired from the top/bottom surfaces and cross sections. Next, the substrates were characterized using an atomic force microscope (AFM, Asylum Research Cypher S) to image surface roughness and features.^[^ ^29^, ^33^, ^97^ ^]^ Three substrates from each group (and two spots from each group, *n* = 6) were evaluated.

##### Isolation of Bone Marrow‐Derived Macrophages

Primary murine bone marrow‐derived macrophages (BMM) were isolated from 5 to 7 week old C57BL/6 black mice (Animal Resources Centre, Australia. Approval for all procedures was obtained from the Griffith University Animal Ethics Committee (DOH/02/16/AEC). The femurs and tibias of mice were aseptically isolated, and adhering tissues were removed. The bone edges were removed, and the marrow cavity was ﬂushed with minimum essential medium alpha (α‐MEM) by slowly injecting at one end of the bone using a sterile needle. Red blood cell (RBC) lysis buffer was treated to remove RBCs. The bone marrow cells were collected with α‐MEM. After washing, the cells were cultured in 100 mm plates with α‐MEM [containing 10% fetal bovine serum (FBS) and 1% penicillin for 1 day at 37 °C in a humidiﬁed atmosphere of 5% CO_2_ in the air with 10 ng mL^−1^ macrophage colony‐stimulating factor (M‐CSF) (Peprotech, NJ, USA)]. On day 2, nonadherent cells were collected and washed with phosphate buffer saline (PBS). After washing, cells were cultured in α‐MEM containing 10% FBS and 1% penicillin for 5 days at 37 °C in a humidiﬁed atmosphere of 5% CO_2_ in the air with 30 ng mL^−1^ M‐CSF.

BMM (M0) were cultured either with lipopolysaccharide (LPS) (Sigma) or interleukin 4 (IL‐4) and interleukin 13 (IL‐13) (Peprotech, NJ, USA) for 48 h to induce an M1 or M2 phenotype, respectively. The M1‐ and M2‐polarized macrophages were then seeded onto Micro‐Ti, Rough‐Ti, TNP‐50, and TNP‐70 surfaces (5 × 10^4^ cells per sample) in tissue culture plates and cultured in Dulbecco's Modified Eagle Medium (DMEM, Gibco, Grand Island, NY) supplemented with 1% PS, 10% FBS, and 30 ng mL^−1^ M‐CSF at 37 °C in a humidified 5% CO_2_ atmosphere for up to 7 days.

##### Cell Proliferation Assay

BMM were seeded onto Micro‐Ti, Rough‐Ti, and TiO_2_ nanopores inside 24 well plates (5.0 × 10^4^/well) and incubated for 7 days. After 2 h, 1, 4, and 7 days of culture, specimens were transferred to fresh plates and cells that adhered to substrates were rinsed with PBS. Later, cell counting kit (CCK‐8) proliferation reagents (Dojindo, Japan) were added to the specimens. After 2 h incubation at 37 °C, reagents were carefully transferred to 96 well plates. The optical density was measured using a microplate reader at a wavelength of 450 nm.

##### Live/Dead Assay

The viability of cells on the various substrates was assessed by live/dead staining. Briefly, BMM cells were seeded at a density of 5.0 × 10^4^ cells/well on the substrates in 24 well plates. After a 24 and 48 h incubation, cells were rinsed with PBS and then incubated with live/dead stain [fluorescein diacetate and propidium iodide] for 30 min at room temperature (RT). Viable cells (green) and dead cells (red) were counted under a confocal laser scanning microscope (CLSM, A1R^+^, Nikon, Japan).

##### Characterization of Cellular Morphology

Cells were fixed after culturing for the times indicated on various implant surfaces for imaging using SEM. Briefly, the implants with cells attached were immersed in fixation buffer [3% glutaraldehyde in 0.1 m cacodylate buffer], after which the cells were washed with cacodylate buffer (0.1 m), treated with osmium tetroxide (1% in cacodylate buffer) and rinsed with ultrahigh quality water. The substrates were then sequentially dehydrated in ethanol (50%–100%) before being treated with hexamethyldisilazane. The substrates were then air‐dried and mounted on SEM holders.^[^ ^30^ ^]^

##### Immunofluorescence Staining for Macrophage Phenotype

Immunofluorescence staining was performed to evaluate the local expression of CD11c (M1 marker) and MR (mannose receptor), known as CD206 (M2 marker). The Mo, M1, and M2 macrophage cells were seeded at a density of 5.0 × 10^4^ cells/well on various Ti/TNP substrates and TCP control and cultured for 1, 4, and 7 days. M1 macrophage was induced by 100 ng mL^−1^ LPS (Sigma, MO, USA) and M2 macrophage by 20 ng mL^−1^ IL‐4 and 20 ng mL^−1^ IL‐13 (Peprotech, NJ, USA) for 2 days from Mo macrophage. These cells were fixed with 3.7% paraformaldehyde for 20 min and permeabilized with 0.05% Triton X‐100 (Thermo Fisher Scientific, Waltham, MA, USA) for 10 min. Later, the cells were stained with primary antibodies for CD11c and CD206 (Santa Cruz Biotechnology Inc., California, USA) overnight and then incubated with secondary antibodies Alexa Fluor 568 goat anti‐rabbit IgG and rabbit anti‐goat IgG‐FITC (Santa Cruz Biotechnology Inc., California, USA) for 1 h. After washing, the substrates were incubated with 4,6‐diamidino‐ 2‐phenylindole dihydrochloride‐DAPI (Invitrogen) for 10 min and observed under a CLSM (A1R^+^, Nikon, Japan).

##### Enzyme‐Linked Immunosorbent Assay

The protein levels of inflammation‐related cytokines (proinflammatory IL‐1β and anti‐inflammatory IL‐10) of BMM cells secreted into cell culture media were measured using a commercially available DuoSet ELISA kit (R&D systems, Minneapolis, MN, USA). BMM cells were cultured at a density of 5.0 × 10^4^ cells/well onto various substrates in 24 well culture plates. At 1, 4, and 7 days of culture, 100 μL of a supernatant sample was collected and incubated plates coated with IL‐1β and IL‐10 antibodies for 2 h at RT. The protein levels of the secreted molecules were measured with a microplate reader at 450 nm according to the manufacturer's instructions.

##### RNA Isolation and Quantitative Real‐Time PCR for Macrophage Phenotype

The PCR was performed at days 1, 4, and 7 to assess the expression of genes associated with M1 and M2 macrophage phenotypes. Total RNA was purified using the TRIZOL reagent (Invitrogen, USA) and RNeasy Plus Mini Kit (Qiagen, CA, USA) according to the manufacturer's instructions. Then, SuperScript IV VILO Master Mix (Invitrogen, USA) was used to prepare complementary DNA (cDNA) according to the manufacturer's protocols. The oligonucleotide primers for arginase 1 (Arg 1), CD163 for the M2 macrophage marker, and CD86, inducible nitric oxide synthase (iNOS) for M1 macrophage markers are listed in Table S1, Supporting Information. Real‐time PCR analysis was performed using KAPA SYBR FAST Master Mix (Kapa Biosystems, Boston, MA), and reactions (LightCycler 480 Real‐Time PCR System, Roche, USA) were run using the following parameters: denaturation at 95 °C for 30 s and 40 cycles of amplification (95 °C for 10 s, 57–60 °C for 20 s, and 72 °C for 1 s). Quantification of gene expression was calculated using the delta Ct method. The expression of the targeted genes was normalized using the geometric average of housekeeping genes glyceraldehyde‐3‐phosphate dehydrogenase (GAPDH). All results were confirmed by repeating the experiment in triplicate. The fold change from TCP substrates at 1 day was set at onefold, and the ratio of the normalized fold change was calculated.

##### Osteoclast Differentiation

For osteoclast differentiation, primary cultures of BMM were used and prepared as detailed in Section 2.3. To stimulate osteoclast differentiation, BMM were seeded at a density of 5 × 10^4^ cells on the substrates in a 24 well culture plate and were cultured in alpha‐MEM supplemented with 10% FBS and 1% penicillin. This was supplemented with RANKL (100 ng mL^−1^) and M‐CSF (30 ng mL^−1^) at 37 °C under 5% CO_2_ for 6 days. Then, the cells were fixed in 3.7% paraformaldehyde for 20 min and permeablized with 0.05% Triton X‐100 for 10 min. After washing, the cells were stained with rhodamine‐conjugated phalloidin (Invitrogen, USA) for 30 min. Actin ring formation was observed using a Zeiss Axiolab fluorescence microscope. In addition, real‐time PCR was performed at 5 days to confirm gene expressions for osteoclast differentiation. Oligonucleotide primers used: tartrate‐resistant acid phosphatase (TRAP), osteoclast‐associated immunoglobulin‐like receptor (OSCAR), nuclear factor of activated T‐cells 1 (NFATc1), and cellular proto‐oncogene (c‐FOS) to assess for osteoclast differentiation marker (Table S1, Supporting Information). Quantification of gene expression was calculated using the delta Ct method. The expression of the targeted genes was normalized using the geometric average of housekeeping genes (GAPDH). All results were confirmed by repeating the experiment in triplicate. The fold change from TCP substrates (positive control) was set at onefold, and the ratio of the normalized fold change was calculated.

##### Cell Culture for Macrophage‐Induced Osteoblasts Differentiation

The MC3T3‐E1 cells (mouse calvaria origin) were used for osteoblast differentiation. The cells were cultured in DMEM supplemented with 10% FBS and 1% antibiotic–antimycotic in a humidified 5% CO_2_ atmosphere. Induction of differentiation to osteoblasts was achieved by culturing the cells with Mo, M1, and M2 macrophage‐conditioned medium on the surface of TiO_2_ nanopores for 4 days and by addition of osteogenic factors [100 nM dexamethasone, 25 μg mL^−1^ l‐ascorbic acid, 10 mM β‐glycerol phosphate (1:1 ratio) in each assay]. The culture medium was changed to 100 nM dexamethasone, 25 μg mL^−1^ l‐ascorbic acid, 10 mM β‐glycerol phosphate after 5 days and the medium was changed every 2 days for 21 days.

##### Alkaline Phosphatase Activity Assay

The alkaline phosphatase (ALP) activity was measured to estimate the osteogenic activity. MC3T3 cells were incubated in osteogenic media at 37 °C with 5% CO_2_ and cultured for 4, 7, 14, and 21 days on Ti/TNP substrates and TCP controls. The cells were seeded at a density of 5 × 10^4^ cells/mL in 24 well plate. After incubation, the cultured cells were washed with PBS, trypsinized, and lysated with RIPA lysis buffer (Sigma, MO, USA) on ice. Aliquots of 150 μL were incubated with 50 μL of p‐nitrophenyl phosphate solution (Sigma, MO, USA) for 30 min at 37 °C. The reaction was stopped by adding 50 μL of 1 N NaOH. The level of p‐nitrophenol production in the presence of ALP was measured by monitoring the light absorbance of the solution at 410 nm using a microplate reader.

##### Alizarin Red S Staining and Assay

Mineralization from the MC3T3‐E1 osteoblast‐like cells was quantified using the alizarin red S (ARS) assay for 1, 2, and 3 weeks. Briefly, cells were fixed by soaking in 3.7% formaldehyde for 15 min. Cells were washed and immersed in ARS solution (pH 4.2) for 30 min at RT with gentle agitation. Then, the solution was removed, and the mineralized matrices were washed with running water. The morphology of the mineralized matrices was observed and imaged using a microscope. To quantify the ARS dye, the stain was solubilized in sodium phosphate buffer (pH 7.0) with 10% w/v cetylpyridinium chloride (Sigma, MO, USA). The absorbance of the solubilized stain was subsequently measured at 540 nm using a microplate reader.

##### RNA Isolation and Quantitative Real‐Time PCR for Osteoblasts Differentiation

The PCR was performed at 1, 2, and 3 weeks to assess the expression of genes associated with osteoblast differentiation. Total RNA was purified using the TRIZOL reagent (Invitrogen, USA) according to the manufacturer's instructions. SuperScript IV VILO Master Mix (Invitrogen, USA) was used to prepare cDNA according to the manufacturer's protocols. Oligonucleotide primers: collagen 1 (COL 1), osteopontin (OPN), ALP, bone morphogenetic protein 2 (BMP‐2), osteocalcin (OCN), bone sialoprotein (BSP), runt‐related transcription factor 2 (RUNX2), and osterix are listed in Table S1, Supporting Information. Real‐time PCR analysis was performed using KAPA SYBR FAST Master Mix (Kapa Biosystems, Boston, MA), and reactions (LightCycler 480 Real‐Time PCR System, Roche, USA) were run using the following parameters: denaturation at 95 °C for 30 s and 40 cycles of amplification (95 °C for 10 s, 57–60 °C for 20 s and 72 °C for 1 s). Quantification of gene expression was calculated using the delta Ct method. The expression of the targeted genes was normalized using the geometric average of housekeeping genes (GAPDH). Experiments were performed in triplicate. The fold change from TCP substrates at 1 day was set at onefold, and the ratio of the normalized fold change was calculated.

##### Statistical Analysis

All experiments were performed in triplicates. All quantitative data are expressed as mean ± statistical differences (SD). The data were analyzed using two‐way ANOVA (three samples per group for all tests, *n* = 3). **p < 0.05, *p < 0.01 were considered as statistically significant.

## Conflict of Interest

The authors declare no conflict of interest.

## Author Contributions

**Ho‐Jin Moon**: Conceptualization (equal); Data curation (equal); Formal analysis (lead); Investigation (lead); Methodology (lead); Writing—original draft (lead); Writing—review & editing (supporting). **Karan Gulati**: Conceptualization (equal); Data curation (lead); Formal analysis (equal); Investigation (equal); Methodology (equal); Project administration (supporting); Resources (supporting); Writing—original draft (equal); Writing—review & editing (lead). **Tao Li**: Data curation (supporting); Formal analysis (supporting); Investigation (equal); Methodology (supporting); Writing—original draft (supporting); Writing—review & editing (supporting). **Corey Stephen Moran**: Data curation (supporting); Resources (supporting); Writing—review & editing (equal). **Sašo Ivanovski**: Conceptualization (equal); Data curation (supporting); Funding acquisition (equal); Methodology (supporting); Project administration (equal); Resources (lead); Supervision (lead); Writing—review & editing (equal).

## Supporting information

Supplementary Material

## Data Availability

The data that support the findings of this study are available in the supplementary material of this article.
