# Supplementary material for: Inflammatory or Reparative? Tuning Macrophage Polarization Using Anodized Anisotropic Nanoporous Titanium Implant Surfaces
Source: Small Sci. 2024 Sep 17;4(10):2400211. doi: 10.1002/smsc.202400211 (PMC11935055; doi:10.1002/smsc.202400211)
Supplement: Supplementary file 1 — Supplementary Material [file SMSC-4-2400211-s001.pdf]

Supporting Information

Inflammatory or Reparative? Tuning Macrophage Polarization using Anodized Anisotropic Nanoporous Titanium Implant Surfaces

Ho-Jin Moon [a,b^], Karan Gulati [c,d^✉], Tao Li [b,e], Corey Stephen Moran[c,d] and Sašo Ivanovski[c,d✉]

^ HJM and KG contributed equally to this work.

[a] Department of Dental Materials, School of Dentistry, Kyung Hee University, Seoul, Republic of Korea

[b] School of Dentistry and Oral Health, Griffith University, Gold Coast, QLD 4222, Australia

[c] The University of Queensland, School of Dentistry, Herston QLD 4006, Australia

[d] Centre for Orofacial Regeneration, Reconstruction and Rehabilitation (COR3), Herston QLD 4006, Australia

[e] Department of Prosthodontics, School of Stomatology, Capital Medical University, Beijing, People's Republic of China

Table S1. Primer sequences for analysed genes.

Functions	Gene	Primer Sequence (5′→3′)
M2 macrophage marker	Arg1	Sense: GTG AAG AAC CCA CGG TCT GT<br>Antisense: GCC AGA GAT GCT TCC AAC TG
	CD163	Sense: CCT GGA TCA TCT GTG ACA ACA<br>Antisense: TCC ACA CGT CCA GAA CAG TC
M1 macrophage markers	iNOS	Sense: CCC TTC AAT GGT TGG TAC ATG G<br>Antisense: ACA TTG ATC TCC GTG ACA GCC
	CD86	Sense: GAG CGG GAT AGT AAC GCT GA<br>Antisense: GGC TCT CAC TGC CTT CAC TC
GAPDH	GAPDH	Sense: GGC ATT GCT CTC AAT GAC AA<br>Antisense: TGT GAG GGA GAT GCT CAG TG
Osteoclast differentiation	TRAP	Sense: CTG GAG TGC ACG ATG CCA GCG ACA<br>Antisense: TCC GTG CTC GGC GAT GGA CCA GA
	OSCAR	Sense: CTG CTG GTA ACG GAT CAG CTC CCC AGA<br>Antisense: CCA AGG AGC CAG AAC CTT CGA AAC
	NFATc1	Sense: CTC GAA AGA CAG CAC TGG AGC AT<br>Antisense: CGG CTG CCT TCC GTC TCA TAG
	c-FOS	Sense: CTG GTG CAG CCC ACT CTG GTC<br>Antisense: CTT TCA GCA GAT TGG CAA TCT C
Osteoblast differentiation	COL	Sense: CGT GGC GAC CAA GGT CCA GT<br>Antisense: AGG GAG ACC CAG AAT ACC GGG AG
	OPN	Sense: CTT TCA CTC CAA TCG TCC CTA<br>Antisense: GCT CTC TTT GGA ATG CTC AAG T
	ALP	Sense: ATC TTT GGT CTG GCT CCC ATG<br>Antisense: TTT CCC GTT CAC CGT CCA C
	BMP2	Sense: CCT TAC CAA AAA TGG AGG CTC A<br>Antisense: GGC TCA GTG TCG TCA TCA TTA AA
	OCN	Sense: CTC TGT CTC TCT GAC CTC ACA G<br>Antisense: GGA GCT GCT GTG ACA TCC ATA C

	BSP	Sense: CGG AGG AGA CAA CGG AGA AG<br>Antisense: GTA AGT GTC GCC ACG AGG CT
	RUNX2	Sense: TCG TCA GCA TCC TAT CAG TTC CCA<br>Antisense: CCG TCA GCG TCA ACA CCA TCA TTC TGG TTA G
	Osterix	Sense: TTG AGG AAG AAG CTC ACT ATG GCT CCA G<br>Antisense: GCT GAA AGG TCA GCG TAT GGC T

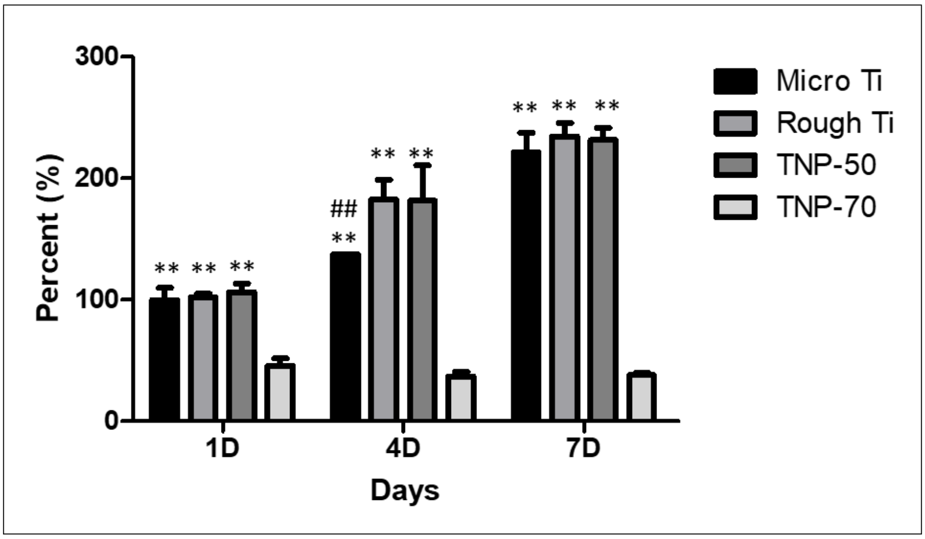

Figure S1. Comparison of macrophage proliferation on various titanium surfaces at 1, 4 and 7 days. ** $p < 0.01$, * $p < 0.05$ significantly different from TNP-70. ## $p < 0.01$, # $p < 0.05$ significantly different from TNP-50. TNP: titania nanopores (50 and 70 nm diameter: TNP-50 and TNP-70).

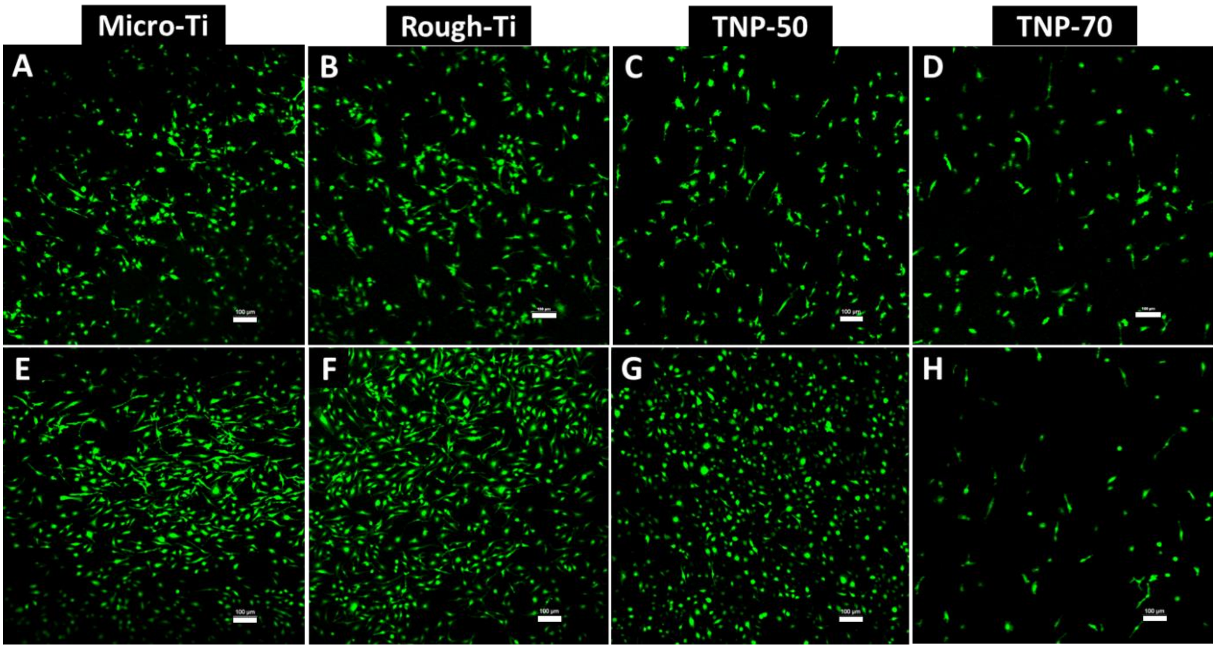

Figure S2. Live and dead assay (primary macrophage) on various Ti substrates. Scale bar = 100 µm. TNP: titania nanopores (50 and 70 nm diameter: TNP-50 and TNP-70).
